# Correlation between duration of edentulism and severity of obstructive sleep apnea in elderly edentulous patients

**DOI:** 10.5935/1984-0063.20210006

**Published:** 2022

**Authors:** Arvind Tripathi, Ashutosh Gupta, Praveen Rai, Piyush Sharma, Suryakant Tripathi

**Affiliations:** 1 Saraswati Dental College, Prosthodontics - lucknow - Uttar Pradesh - India.; 2 Dental College Azamgarh, Orthodontics - Azamgarh - Uttar Pradesh - India.

**Keywords:** Obstructive Sleep Apnea, Edentulous, Mandibular Advancement Device

## Abstract

**Objectives:**

To investigate the correlation between duration of edentulism and severity of obstructive sleep apnea in elderly edentulous patients.

**Material and Methods:**

1,017 patients aged 55-65 years, with a history of edentulism of 12-60 months were screened. Detailed history of tooth loss and period of edentulism was recorded for the 414 patients who tested positive for OSA (obstructive sleep apnea). Complete dentures were prepared for each patient and they were trained to use the dentures as a mandibular advancement device (MAD) during sleep at night. Apnea-hypopnea index (AHI) data at pre-treatment, six months and one-year post-treatment time intervals was recorded. A correlation between the period of untreated edentulism and severity of OSA and improvement post-treatment was derived in this study.

**Results:**

Mean duration of edentulism was 12.14±2.57 months and mean AHI was 16.62±13.24. For every three month increase in the duration of edentulism (after initial 6 months of total tooth loss), there was a statistically significant increase in severity of OSA. Patients who are edentulous for more than 15 months are increasingly vulnerable to OSA.

**Discussion:**

Severity of OSA in afflicted long-term edentulous patients was in direct relation to the period of untreated edentulism and regressed likewise with concomitant denture wear and mandibular advancement during sleep at night. Early prosthetic rehabilitation of edentulous patients is imperative to obviate morbidity of OSA.

## INTRODUCTION

Edentulism or loss of all permanent teeth^1^ is the final outcome of “multifactorial process involving various biological and non-biological factors related to dental procedures”^2^. World Health Organization (WHO) considers edentulism as physical impairment, disability and a handicap^3-5^. Epidemiological studies have provided a variable country/area specific data on its incidence ranging from 3 to 80%^2,6^. Some researchers have predicted a rise in edentulism in future^7^ due to global increase in life expectancy while most researchers^8-10^ foresee a definite decline in complete tooth loss because of the global impact of dentistry and prosthodontics. Yet, edentulism has not been eliminated and has been associated with multitude of comorbidities such as malnutrition, osteoporosis, obesity, cardiovascular diseases, diabetes, rheumatoid arthritis, respiratory afflictions including chronic obstructive pulmonary disease (COPD), cancer, cognitive disorders, and even mortality^2,11,12^.

Following edentulism, various esthetic and soft tissue profile changes occur that include loss of vertical dimension of occlusion^12-15^, reduction of lower facial height, antero-superior rotation of mandible^2,16,17^. Edentulism is also accompanied by various functional and sensory deficiencies of the stomatognathic apparatus^18^. Changes in mandibular position, oral hypo-innervation, decreased neuromuscular coordination^12,19^, heightened neuromuscular impairment favoring upper airway collapse by deactivation of pharyngeal dilator muscles in response to stimuli^20-24^, prominent alterations in upper airway size, its elasticity and function^12-15^, increased upper airway collapsibility^25^, decreased retropharyngeal space^26^, reduced tonicity of the pharyngeal musculature^27,28^, increased type I collagen in the extracellular matrix of the superior pharyngeal constrictor muscle^29^; interplay of all these factors predisposes to the development of obstructive sleep apnea (OSA) of varying severity.

In aged individuals, upper airway reflex sensitivity and genioglossus muscle response to hypoxia is reduced, leading to depleted upper airway muscle function at onset of sleep and a more collapsible airway. A reduction in lumen and lengthening of the pharyngeal airway leads to increased airway resistance and a predisposition to airway collapse^30^. Wheatley and Amis (1998)^31^ found a narrowing of the upper airway laterally, with a thickening of the lateral pharyngeal wall in elderly OSA patients. Deranged upper airway muscle tonicity triggered a coordinated and increased muscle activity to compensate for the anatomically narrow and more collapsible airway while awake, which reduced at sleep leading to pharyngeal collapse and elevated OSA symptoms.

Sanders et al. (2016)^32^ found edentulism to be an independent risk factor for OSA. Heidsieck et al. (2016)^26^ cited obstructive sleep apnea as a major medical affliction, affecting 15-30% adult males and 5-15% adult females^33,34^ and a progenitor of several systemic complications^28,35-39^ along with a higher risk of mortality. Tripathi et al. (2019)^12^ reported a prevalence of OSA in 40.94% of the edentulous population (32.03% in males and 8.91% in females). Zou et al. (2016)^40^ found that about 31% of the edentulous population was at high risk of OSA and, despite gender differences in airway anatomy, both males and females were equally vulnerable. Won and Guilleminault (2015)^41^ found that though the incidence of OSA was less in females yet the morbidity was more marked and severe compared to males. Wimms et al. (2016)^42^ found that women were able to stabilize their breathing better than males during sleep, leading to less severe apneas and minimal desaturation. This, along with lesser upper airway collapsibility led to milder OSA symptoms in them.

Bucca et al. (1999, 2006)^16,17^ reported that complete tooth loss worsened OSA and that nocturnal wearing of dentures improved the apnea-hypopnea index (AHI) of such patients. Since complete tooth loss augments upper airway collapse and could cause OSA, hypothetically prosthodontic service by way of complete dentures could reverse the malady. However, nocturnal wearing of complete dentures to prevent upper airway collapse has yielded variable results^40,43-48^. With an increase in average human life expectancy, prosthodontic rehabilitation of edentulous patients has come to be both a major treatment need and a global comprehensive effort. Yet, varied reasons viz. lack of access to a treatment facility, economic constraints, and lack of old age support, may restrict or delay rehabilitation and potentiate symptoms of upper airway obstruction. No literature is available to correlate time elapsed since total tooth loss and severity of OSA. The present study attempted to correlate the period of untreated edentulism on severity of OSA in elderly patients. Our null hypothesis was that there would be no correlation between period of edentulism and severity of OSA in different age groups.

## MATERIAL AND METHODS

Four hundred and fourteen patients, from a rural setting, aged 50-65 years, with a history of edentulism of 12-60 months, in need of complete denture rehabilitation brought to the Prosthodontics Clinic, Saraswati Dental College & Hospital, Lucknow, India between March 2012 and March 2019 participated in this study. The study protocol was approved by the Institutional Human Ethical Committee and Institutional Research & Development Committee and the research was conducted in full accordance with ethical principles, including the world medical association declaration of Helsinki. Primary screening of these patients was done according to the inclusion and exclusion criteria delineated for the study. These patients did not have access to any dental healthcare facility, because of their remote location and other logistic barriers and were brought to the clinics by our outreach team.

**Figure 1:**
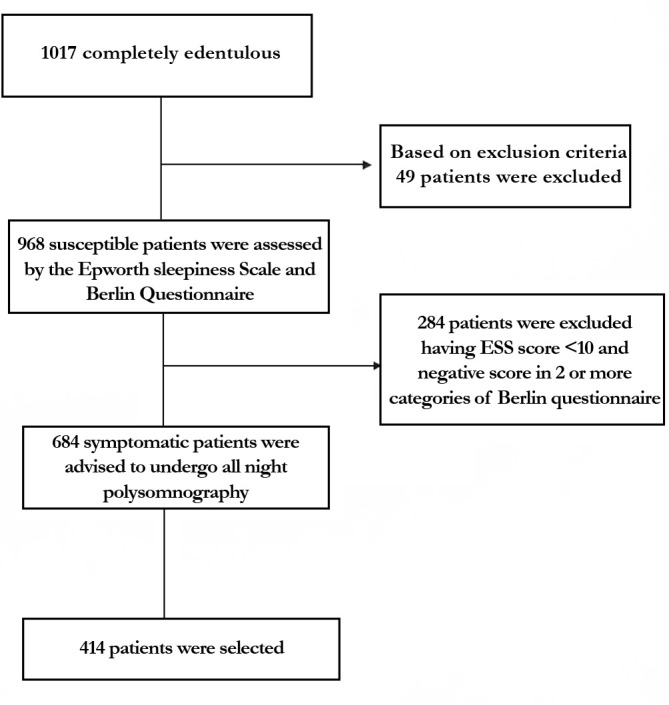
Flowchart for patient selection.

**Figure 2:**
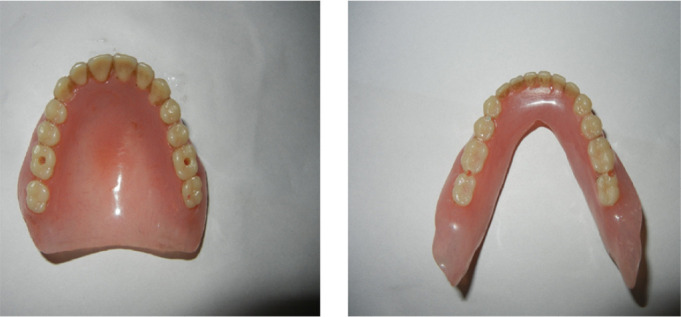
Complete dentures with hole at molar region.

**Figure 3:**
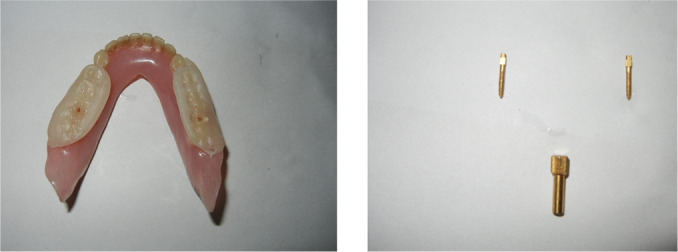
Mandibular denture with soft liner wafer and screw to immobilize the denture and soft liner.

**Figure 4:**
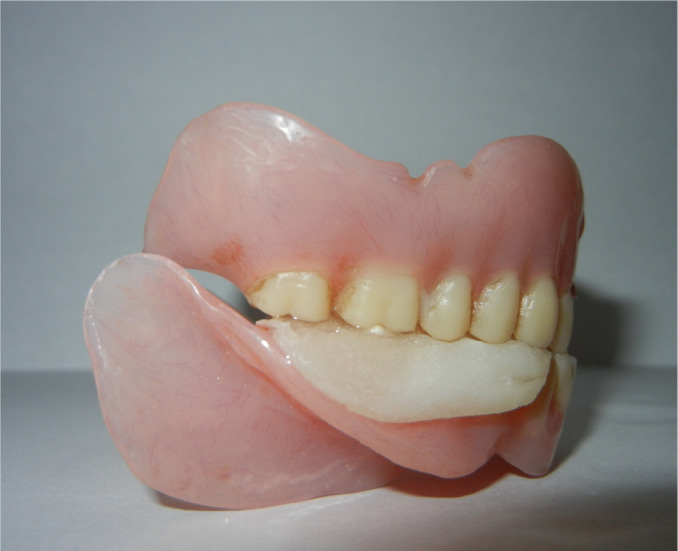
The 3 unit modified mandibular advancement device.

**Figure 5:**
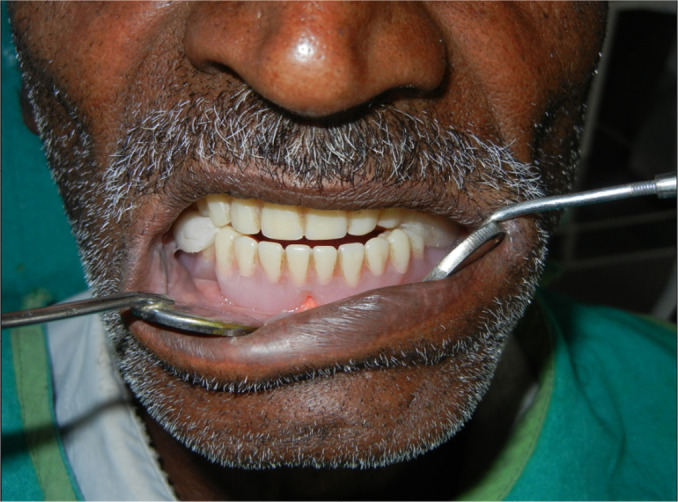
The intra oral view of modified mandibular advancement device.

The inclusion criteria were elderly non-obese (basal metabolic index 18-24kg/m^2^) patients 50-65 years of age, completely edentulous and unrehabilitated since 12-60 months, having a prosthodontic diagnostic index classification (ICD9-CM diagnostic code) score of 525.41. The exclusion criteria were: patients above 65 years of age to rule out the possible inclusion of patients with central sleep apnea, TMJ disorders including pain, significant joint crepitation, restricted mouth opening, chronic obstructive pulmonary disease, and asthma. This primary screening yielded a cohort of 1,017 susceptible patients. They were assessed for fragmented sleep and sleep disordered breathing by the Epworth sleepiness scale, Berlin questionnaire, and the STOP-BANG questionnaire. 684 reported symptomatic and were subjected to all night polysomnography (PSG) (EMBLA-7000 Cogent Technologies). 324 patients were found to be afflicted with OSA, and were classified according to the severity of the affliction (mild, moderate, and severe) based on the assessment data on apnea-hypopnea index (AHI). The flow chart for patient selection is summarized in Figure 1. A detailed history of tooth loss and the period of untreated edentulism was recorded. Complete dentures with fixtures^49,50^ were fabricated and provided to these 324 patients they were taught to convert and use these as mandibular advancement device (MAD) during sleep at night (Figures 2, 3, 4 and 5). Data on the period of edentulism and pre-treatment and one year post treatment apnea-hypopnea index (AHI) was recorded. Data analysis was done using Statistical Package for Social Sciences version 24.0. ANOVA, Tukey HSD test and linear regression and inferences were drawn. A correlation between the period of untreated edentulism and severity of OSA and improvement post-treatment was derived in this study. A ‘*p*’ value less than 0.05 was considered statistically significant.

## RESULTS

A total of 414 subjects (342 male, 72 female) completed the entire assessment. Majority were males (82.6%). Age of patients ranged from 50 to 65 years. Mean age was 60.11±3.16 years. Duration of edentulism ranged from 6 to 18 months. A total of 64 (15.5%) patients were edentulous for 6-9 months, 149 (36.0%) were edentulous for 10-12 months, 178 (43.0%) were edentulous for 13-15 months and 23 (5.6%) were edentulous for >15 months. Mean duration of edentulism was 12.14±2.57 months. On polysomnographic assessment, 90 (21.7%) had AHI<5 (minimal/no OSA), 109 (26.3%) had AHI 5-15 (mild OSA) and 177 (42.8%) had AHI 15-24 (moderate OSA). There were 38 (9.2%) with AHI>30. Mean AHI was 16.62±13.24 (range 1-77) (Table 1).

**Table 1:** Patient characteristics, duration of edentulous and AHI scores.

SN	Characteristic		Statistic	
1.	Gender			
	Male		342 (82.6%)	
	Female		72 (17.4%)	
		**Total**	**Males (n=342)**	**Females (n=72)**
2.	Mean age±SD (range) in years	60.11±3.16 (50-65)	60.65+3.06 (50-65)	57.56+2.25* (55-65)
3.	Duration of edentulousness			
	6-9 months	64 (15.5%)	56 (16.4%)	8 (11.1%)
	10-12 months	149 (36.0%)	128 (37.4%)	21 (29.2%)
	13-15 months	178 (43.0%)	138 (40.4%)	40 (55.6%)
	>15 months	23 (5.6%)	20 (5.8%)	3 (4.2%)
	Mean±SD (range) in months	12.14+2.57 (6-18)	12.10+2.60 (6-18)	12.33+2.44 (6-18)
4.	AHI score			
	None (<5)	90 (21.7%)	35 (10.2%)	55 (76.4%)
	Mild (5-15)	109 (26.3%)	97 (28.4%)	12 (16.7%)
	Moderate (15-30)	177 (42.8%)	173 (50.6%)	4 (5.6%)
	Severe (>30)	38 (9.2%)	37 (10.8%)	1 (1.4%)
	Mean±SD (range)	16.62+13.24 (1-77)	18.96+13.04 (1-77)	5.50+7.16 (1-52)*

* Independent samples ‘t’-test used.

Proportion of patients with moderate to severe grade of OSA showed an incremental trend with increasing duration of edentulism. 49% patients with period of edentulism more than 12 months were found to suffer from OSA. 58.8% of patients with mild OSA, 40.6% of patients with moderate OSA and all severe OSA patients (100%) were edentulous for more than 12 months. Statistically, this difference was significant (*p*<0.001) (Table 2). On evaluating the overall trend of mean AHI values and duration of edentulism, a direct linear relationship between these two entities was observed (r=0.539; *p*<0.001) (Table 3).

**Table 2:** Association between grade of OSA and duration of edentulism.

Grade of OSA	Duration of edentulism			
	6-9 months	10-12 months	13-15 months	>15 months
Overall (n=414)				
None/Minimal (n=90)	41 (45.6%)	22 (24.4%)	27 (30.0%)	0 (0.0%)
Mild (n=109)	15 (13.8%)	30 (27.5%)	61 (56.0%)	3 (2.8%)
Moderate (n=177)	8 (4.5%)	97 (54.8%)	70 (39.5%)	2 (1.1%)
Severe (n=38)	0 (0.0%)	0 (0.0%)	20 (52.6%)	18 (47.4%)
H=100.76; p<0.001 (Kruskal-Wallis H test)				

Percentages have been calculated row-wise.

**Table 3:** Comparison of Mean AHI Scores among patients with different duration of edentulism.

Duration (in months)	N	Mean	SD	Minimum	Maximum
Overall (n=414)					
A. 6-9 months	64	5.02	4.51	1	18
B. 10-12 months	149	16.17	8.19	1	30
C. 13-15 months	178	18.62	14.82	1	77
D. >15 months	23	36.35	13.58	14	55
Total	414	16.62	13.24	1	77
F=46.245; p<0.001 (ANOVA); r=0.465; p<0.001; (Pearson correlation coefficient).					

Average AHI values derived from all night polysomnography after 1 year of regular MAD wear are presented in Table 4. Patients with period of edentulism 6-9 months recorded a decline in AHI from 5.02 to 3.6 (*p*<0.01). Patients with period of edentulism 10-12 months recorded a decline in AHI from 16.17 to 4.3 (*p*<0.01). Patients with period of edentulism 13-15 months recorded a decline in AHI from 18.62 to 4.9 (*p*<0.01) and patients who were rehabilitated after >15 months of edentulism showed a regression from 36.35 to 5.3 in AHI (*p*<0.01).

**Table 4:** Change in AHI at 1 year after insertion of MAD.

S. No.	Period of edentulism	Baseline AHI	Post treatment AHI	Tukey HSD *p*-value
1.	6-9 months	5.02	3.6	0.0010053
2.	10-12 months	16.17	4.3	0.0010053
3.	13-15 months	18.62	4.9	0.0010053
4.	>15 months	36.35	5.3	0.0010053

On multivariate assessment (linear regression) (Table 5) with post appliance use (denture and MAD) AHI as dependent variable and period of edentulism as predictor, ANOVA showed a highly significant correlation in change of AHI value after using MAD.

**Table 5:** Linear regression equation.

Coefficients^a^						
Model		Unstandardized coefficients		Standardized coefficients	t	Sig.
		B	Std. Error	Beta		
1	(Constant)	1.949	.209		6.923	.000
	Independent_x	.216	.012	.851	18.730	.000

a Dependent variable: Dependent_y.

The mathematical formula derived was using regression analysis, linear regression equation was derived:

Y=1.449+0.216x

Y is dependent variable, i.e., AHI value obtained after using MAD for 12 months.

x is independent predictor, initial period of edentulism.

## DISCUSSION

Various studies conducted by Bucca et al. (1999, 2006)^16,17^, Oksayan et al. (2015)^47^, and Zou et al. (2016)^40^ have reported a correlation between edentulism and occurrence of OSA. However, no literature was available on a possible correlation between duration of edentulism and severity of OSA.

The sample size of the present study after excluding the dropouts was 414. In previous studies done by Bucca et al (2006)^17^, Oksayan et al. (2015)^47^, and Zou et al (2016)^40^ the sample size was 48, 42, and 400, respectively. As no previous data on a possible correlation on duration of untreated edentulism and OSA severity was available, hence, sample size calculation could not be done and convenience-sampling method was used for this study.

The age range of sample was between 50-65 years as most of the edentulous patients seeking rehabilitation are usually above 50 years of age. The duration of edentulism for the study sample ranged between 6 to 18 months. As maximum and rapid changes in stomatognathic apparatus occur in the initial six months following extraction of teeth, so patients who were edentulous for less than 6 months were excluded. Lack of awareness about the disability in edentulous state, inability to seek treatment due to social and economic dependence on the younger caregivers and ultimately, a stoic acceptance of altered lifestyle; all contribute to delayed attempts at prosthetic rehabilitation.

The maximum extent of the span of edentulism was taken as 18 months as most of the patients were able to seek prosthodontic rehabilitation only after a year following total tooth loss, when they perceive significant systemic and esthetic issues consequent to edentulism. Our study cohort was derived from edentulous patients from an urban setting, visiting the outpatient clinic of the department of prosthodontics. All these patients had some level of education and were well aware of the perils of edentulism, so, we did not encounter any patient with a span of untreated edentulism of more than 18 months. Following edentulism, a cascade of events occurs that predispose to development of OSA. Many of these changes such as loss of vertical dimension, rotation of mandible, retracted tongue position, etc., are reversible with prosthodontic rehabilitation. However, neuronal changes such as partial neurodegeneration in the oral cavity and neuromuscular in-coordination may or may not revert to the same extent (dentate stage) even after prosthodontic rehabilitation^51^.

In the present study, it was found that severity of OSA escalated in a direct proportion to the period of edentulism. For every three months increase in duration of edentulism (after initial 6 months of total tooth loss), there was a statistically significant increase in severity of OSA. Around 35% patients who were edentulous for 6-9 months developed OSA and as the duration of edentulism increased to 10-15 months, 85% patients were found to suffer from OSA of varying intensity. Beyond 15 months of edentulism, all patients suffered from OSA, with nearly 78% in the severe form. Thus, our null hypothesis was rejected. It seems pertinent that early prosthodontic rehabilitation may prevent the onset/reduce the severity of OSA in the geriatric edentulous population. However, through this preliminary study, the choice/type of prosthodontic rehabilitation cannot be predicted.

In the present study, AHI value 1 year after the use of MAD was in direct liner relationship with initial period of edentulism. The less was the initial period of edentulism the better was the effect of MAD. Thus, it can be said that early prosthetic rehabilitation may serve to prevent the regressive changes occurring in the oral cavity and pharyngeal airway space. However, the available literature is ambiguous regarding the nocturnal use of conventional complete dentures in elderly sleep apnea patients. Almeida et al. (2012)^43^, Chen et al. (2017)^44^ found that OSA patients experience more apneic events and increase in AHI with complete dentures in place during sleep. In contrast, Arisaka et al. (2009)^45^ found that in some patients, AHI improved by wearing complete dentures during sleep whereas in some patients, AHI increased and worsened OSA. Erovigni et al. (2005)^46^ and Oksayan et al. (2015)^47^ also reported a positive effect on AHI values and OSA with use of dentures.

It is important to understand that dentures only simulate teeth but they cannot duplicate the intricate anatomic and functional relationships that exist between natural teeth and surrounding tissues. In a study conducted by Tripathi et al. (2014)^50^, it was found that if complete denture prosthesis is modified to serve as mandibular advancement device, then, it is effective in lowering AHI and improves OSA. If the complete denture position is not fixed in supine position during sleep, then the mandibular denture would actually add on to the total weight of the mandible and further promote its backward slide, thus promoting a probability of pharyngeal airway obstruction.

The TMJ must be in healthy state for proper functioning of this MAD. Since appliance requires forward positioning of the mandible, hence any structural deformity will affect the normal range of motion of the TMJ. In our previous study, we found no effect on TMJ with this appliance. However, in certain cases, altered maxillomandibular relationship (due to mandibular advancement) may lead to impingement of dental prosthesis in newer areas and cause fresh denture soreness. In this event, such spots must be identified and the denture suitably modified to prevent any impingement^50^.

The limitations of the present study include inability to include elderly patients with duration of edentulism exceeding 18 months.

## CONCLUSION

The results of this study indicate that long-term edentulism foretells greater morbidity than a mere depletion of esthetics, speech, mastication, and nutrition. It can potentiate OSA and consequent life threatening multiple organic dysfunction.
